# Supplementary material for: TBC1D23 mediates Golgi-specific LKB1 signaling
Source: Nat Commun. 2024 Feb 27;15:1785. doi: 10.1038/s41467-024-46166-2 (PMC10899256; doi:10.1038/s41467-024-46166-2)
Supplement: Supplementary file 1 — Supplementary Information [file 41467_2024_46166_MOESM1_ESM.pdf]

Supplementary Information for:

TBC1D23 mediates Golgi-specific LKB1 signaling

Yingfeng Tu[1,7], Qin Yang[1,7], Min Tang[1,7], Li Gao[2], Yuanhao Wang[3], Jiuqiang Wang[4,5], Zhe Liu[1],

Xiaoyu Li[1], Lejiao Mao[1], Rui zhen Jia[1], Yuan Wang[2], Tie-shan Tang[4], Pinglong Xu[6], Yan Liu[3],

Lunzhi Dai[2], Da Jia[1]

Correspondence should be addressed to D.J. (e-mail: jiada@scu.edu.cn)

The PDF file includes:
Supplementary Figures 1 to 7
Supplementary Table 1

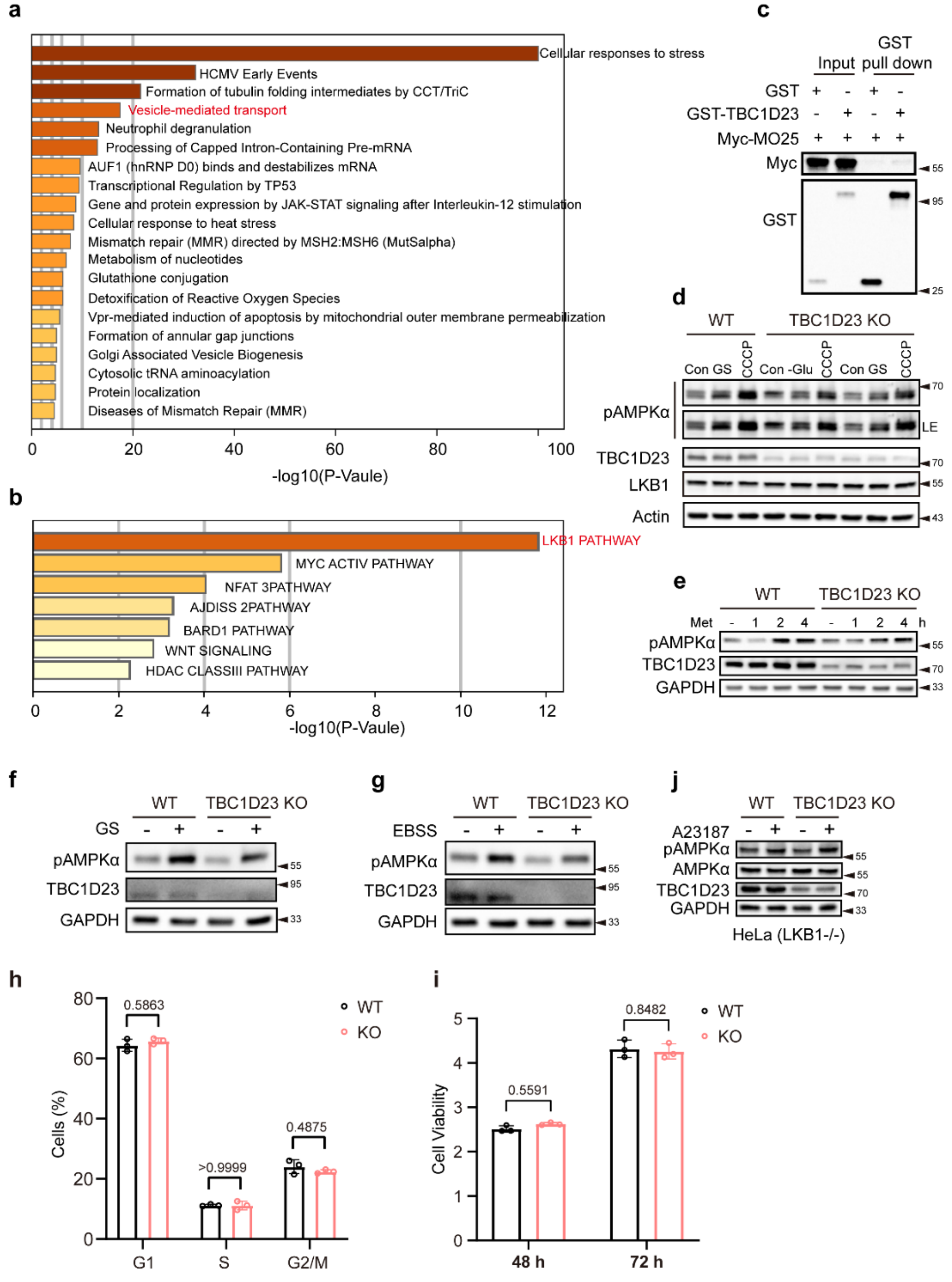

Fig. S1 LKB1 is a novel TBC1D23 interactor that is required for AMPK activation upon energy stress. a and **b**, Analysis of TBC1D23-interacting proteins. Reactome gene sets (**a**) and canonical pathway (**b**) analysis of precipitated proteins by GST-TBC1D23. **c**, the weak interaction between TBC1D23 and MO25. HEK293T cells were transfected with GST-TBC1D23 and Myc-

MO25, co-transfection of GST-vector and Myc-MO25 as the negative control, followed by precipitation with GST beads and immunoblotting with antibodies against GST and Myc. **d** and **e**, TBC1D23 deficiency results in impaired AMPK activation. WT and TBC1D23 KO pool HepG2 cells were subjected to glucose starvation, CCCP treatment (**d**) or treated with 5 mM metformin for indicated time (**e**). Cells were collected and analyzed with immunoblotting. **f** and **g**, TBC1D23 deficiency results in attenuated AMPKα activation. WT and TBC1D23 KO pool HepG2 cells were starved with glucose (**f**) or EBSS (**g**) for 2 h, harvested and immunoblotted with pAMPKα, TBC1D23 and GAPDH. **h**, Cell cycle analysis of WT and TBC1D23 KO cells. WT and TBC1D23 KO HepG2 cells were collected and cell cycle profiles were determined by FACS. **i**, WT or TBC1D23 KO HepG2 cells were cultured for indicated time, and cell viability was assessed using the CCK8 assay and normalized to that of 0 h. **j**, CaMKK2-AMPK pathway remains intact in TBC1D23 deficient cells. WT and TBC1D23 KO HeLa cells were treated with or without 2 μM A23187 for 30 min, cells were then lysed and analyzed by indicated immunoblotting. Experiments **c**–**i** were performed in triplicate. Results are presented as mean ± SD. P values were calculated by two-way ANOVA followed by Sidak's multiple comparisons test.

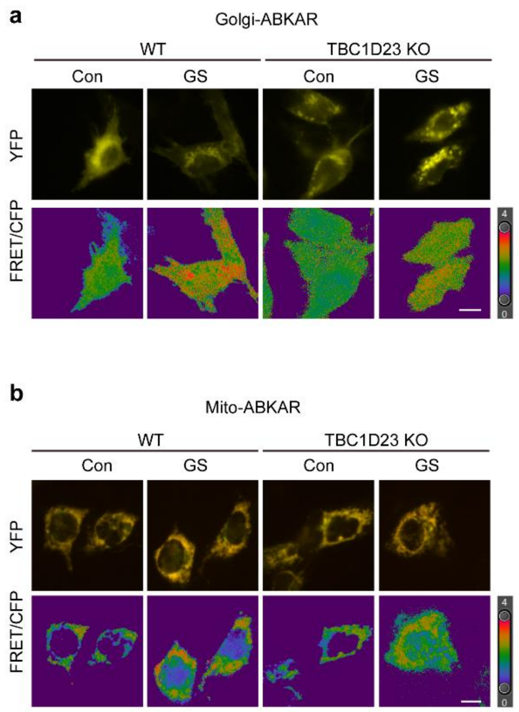

Fig. S2 Deletion of TBC1D23 specifically impairs Golgi-AMPK activation. Representative FRET image of Golgi-ABKAR and Mito-ABKAR. WT and TBC1D23 KO HepG2 cells transiently transfected with Golgi-ABKAR (**a**) or Mito-ABKAR (**b**) were incubated with or without glucose-free medium for 2 h and 4 h, respectively. And the FRET/CFP ratio was measured. Representative YFP images (upper); representative pseudocolor images of FRET/CFP ratio show the FRET response (lower). Scale bar, 10 μm.

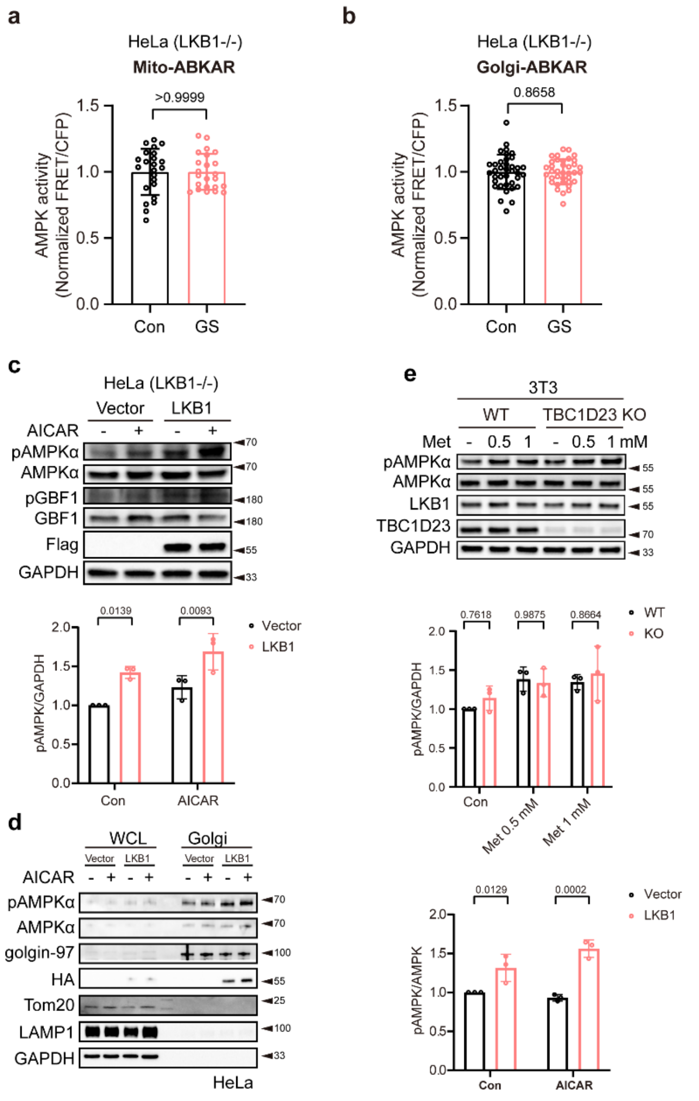

Fig. S3 LKB1 is responsible for Golgi-AMPK activation upon energy stress. a and **b**, HeLa cells transiently transfected with Mito-ABKAR (**a**) or Golgi-ABKAR (**b**) were incubated with glucose-free medium for 2 h and 4 h, respectively. The FRET/CFP ratio was measured and shown relative to HeLa cells incubated with DMEM. (**a**), Con: n=23 cells; GS: n=23 cells. (**b**), Con: n=37 cells; GS: n=38 cells. n indicates pooling cells from one replicate**. c**, HeLa cells transiently transfected with vector or LKB1 were treated with 2 mM AICAR for 2 h, harvested, and immunoblotted with indicated antibodies. The graph shows the levels of pAMPK quantified by densitometry using Image

J software and normalized to GAPDH. Values of pAMPK to GAPDH were shown relative to the ratio of pAMPK to GAPDH in untreated HeLa cells transfected with vector. n=3 independent experiments. **d**, HeLa cells stably transfected with plvx-vector or plvx-LKB1-HA were treated with 2 mM AICAR for 2 h, harvested and subjected to Golgi extraction. Whole cell lysate and Golgi fractions were analyzed by immunoblotting with indicated antibodies (left). Golgin-97, Tom20 and LAMP1 were used as markers for the Golgi, mitochondria and lysosome, respectively. The graph shows the levels of pAMPK in Golgi fraction quantified by densitometry using Image J software and normalized to AMPK. Values of pAMPK to AMPK were shown relative to the ratio of pAMPK to AMPK in untreated HeLa cells stably transfected with plvx-vector (right). n=3 independent experiments. **e**, WT and TBC1D23 KO 3T3 cells were treated with metformin at concentrations indicated for 2 h. Cells were collected and analyzed by immunoblotting with indicated antibodies (upper). The graph shows the levels of pAMPK quantified by densitometry using Image J software and normalized to GAPDH. Values of pAMPK to GAPDH were shown relative to the ratio of pAMPK to GAPDH in untreated WT 3T3 cells (lower). n=3 independent experiments. Results are presented as mean ± SD. P values were determined using an unpaired two-tailed t test (**a** and **b**) or two-way ANOVA followed by Sidak's multiple comparisons test (**c,d** and **e**).

a

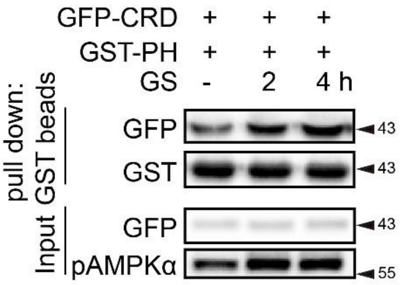

b

LKB1/TBC1D23 interaction slimulated by AMPKα

LKB1	Condition	Enhanced TBC1D23 binding	AMPK activation
FL	Con	Yes	Yes
	GS	Yes	Yes
CRD	Con	NO	NO
	GS	Yes	Yes (by endogenous LKB1)

Fig. S4 The interaction between TBC1D23 and LKB1 is dynamically regulated upon energy stress. a, GST-pull down assay showing the enhanced interaction between TBC1D23 and LKB1 upon glucose starvation. HEK293T cells transfected with GFP-LKB1 CRD were subjected to glucose starvation for indicated time before harvest. Cell lysates were incubated with GST-TBC1D23 PH immobilized on GST beads, and bound GFP-LKB1 CRD and purified GST-TBC1D23 PH were detected by immunoblotting. **b**, summary of effects of AMPKα on the interaction between TBC1D23 and LKB1 under physiological conditions and upon energy stress.

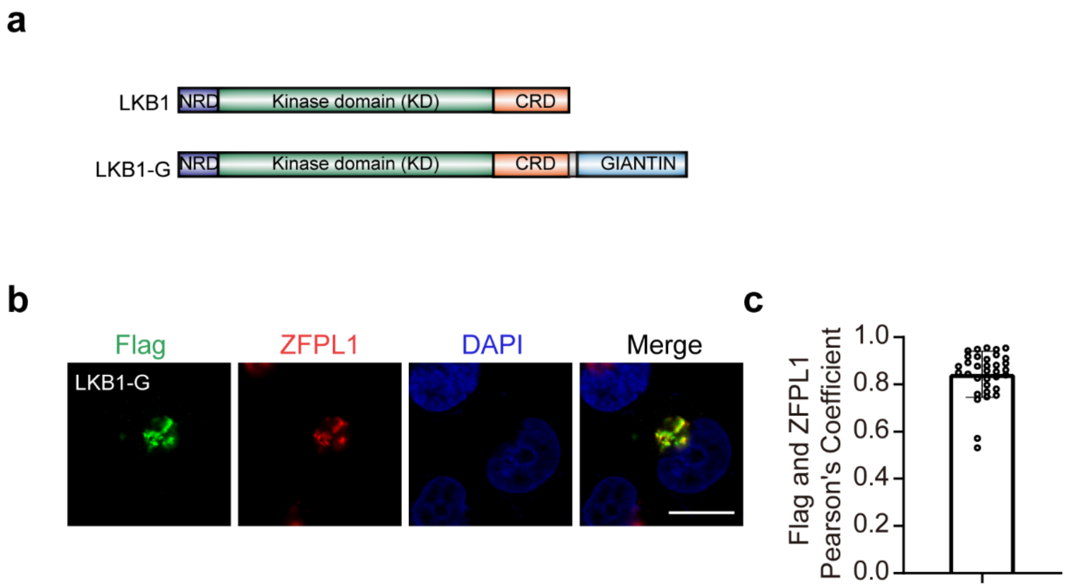

Fig. S5 Golgi-targeted expression of LKB1. a, Schematics of the LKB1-Giantin (LKB1-G) chimaera. The chimaera contains full length of LKB1, a linker region (GGSGGSGGS), and aa3,131-3,259 of GIANTIN. **b**, Confocal micrographs showing that the LKB1-G construct targets the Golgi in HeLa cells. Scale bar: 10 μm. **c**, bar graph represents quantitation of Flag co-localization with ZFPL1 (Golgi marker). Each dot represents Pearson's correlation coefficients from one cell. n=34 cells. n indicates pooling cells from one replicate. Similar results were obtained in three independent experiments. Scale bar, 10 μm.

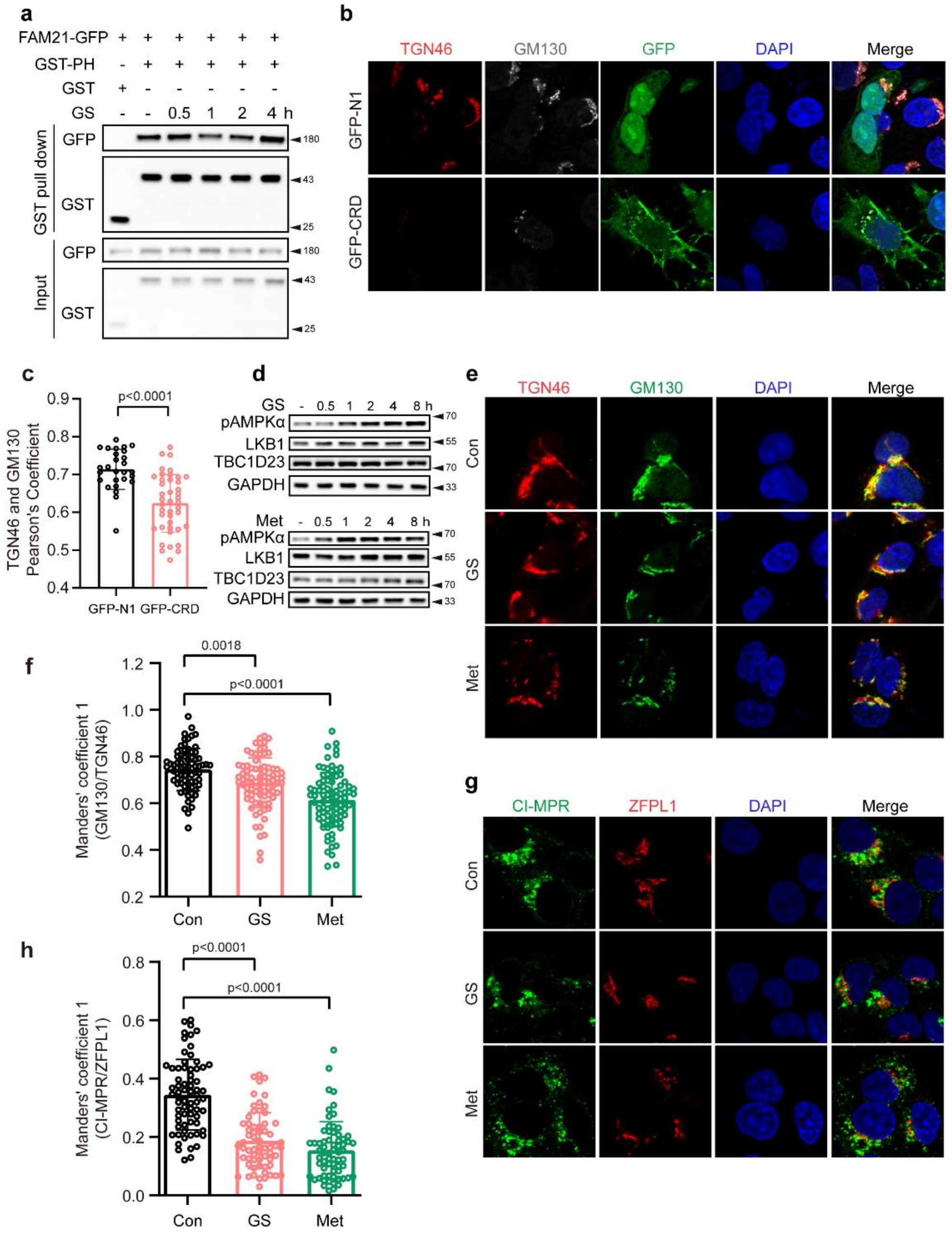

Fig. S6 TBC1D23-mediated AMPK activation downregulates endosome-to-TGN trafficking.

a, GST pull-down experiments showing the dynamic interaction between TBC1D23 and FAM21 upon glucose starvation. HEK293T cells were transfected with FAM21-GFP and GST-TBC1D23 PH. Control cells were co-transfected with GST-vector and FAM21-GFP. 24 h later, cells lysates

were subjected to glucose starvation for indicated time and precipitation with GST beads, followed by immunoblotting with indicated antibodies. **b**, Confocal imaging of HeLa cells transfected with GFP-LKB1 CRD or empty vector for 24 h. Cells were stained with antibodies against TGN46 and GM130. Pearson's correlation coefficients of TGN46 and GM130 were calculated using image J. **c**, Bar graph represents quantitation of TGN46 co-localization with GM130. Each dot represents Pearson's correlation coefficients from one cell. GFP-N1: n=27 cells; GFP-LKB1 CRD: n=42 cells. n indicates pooling cells from one replicate. Scale bar, 10 μm. **d**, Immunoblot analysis showing that glucose starvation and metformin induces AMPK activation. HepG2 cells treated with glucose starvation (upper) or metformin (lower) for indicated time before harvest and immunoblotted with indicated antibodies. **e**, HepG2 cells were treated with metformin (10 mM, 4 h) or medium deprived of glucose for 4 h, then fixed, and labeled with anti-TGN46 (red) and GM130 (green) antibodies. **f**, Quantitation of TGN46 co-localization with GM130 in cells as treated in e. Each dot represents Manders' coefficient 1 (GM130 overlapping with TGN46) from one cell. Con: n=72 cells; GS: n=84 cells; Met: n=86 cells. n indicates pooling cells from one replicate. Scale bar, 10 μm. **g**, HepG2 cells were treated with metformin (10 mM, 4 h) or medium deprived of glucose for 4 h, then fixed, and labeled with anti-CI-MPR (green) and ZFPL1 (red) antibodies. **h**, Quantitation of CI-MPR co-localization with ZFPL1 in cells as treated in **g**. Each dot represents Manders' coefficient 1 (CI-MPR overlapping with ZFPL1) from one cell. Con: n=69 cells; GS: n=63 cells; Met: n=66 cells. n indicates pooling cells from one replicate. Scale bar, 10 μm. Experiments a–h were performed in triplicate. Results are presented as mean ± SD. P values were determined by an unpaired two-tailed t test (**c**) or one-way ANOVA followed by Dunnett's multiple comparisons test (**f** and **h**).

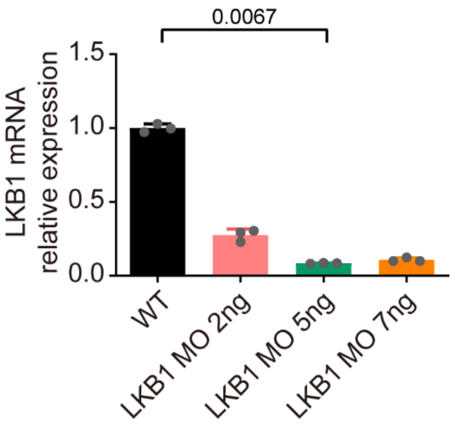

Fig. S7 LKB1 MO efficiently reduces the expression of LKB1 in zebrafish embryos. RT-PCR showing that injection of LKB1 MO (5 ng) effectively decreased the mRNA level of LKB1. All injections were performed at the one cell stage of zebrafish development. ACTIN was used as a loading control. p values were calculated by Kruskal-Wallis test.

Supplementary Table 1. Statistical data.

Fig. 1			
Fig.1f	siNC	Con	1±0, n=3
		GS 2 h	1.75±0.35, n=3
		GS 4 h	1.48±0.25, n=3
		CCCP	1.31±0.10, n=3
	SiTBC1D23	Con	1.00±0.34, n=3; p >0.9999, compared to siNC con
		GS 2 h	0.91±0.23, n=3; p= 0.0010, compared to siNC GS 2 h
		GS 4 h	0.92±0.07, n=3; p= 0.0268, compared to siNC GS 4 h
		CCCP	0.80±0.17, n=3; p= 0.0471, compared to siNC CCCP
Fig. 1g	WT	Con	1±0, n=3
		GS 1 h	1.78±0.27, n=3
		GS 2 h	1.76±0.14, n=3
	KO	Con	0.87±0.13, n=3; p= 0.7178, compared to WT con
		GS 1 h	1.01±0.21, n=3; p= 0.0003, compared to WT GS 1 h
		GS 2 h	1.13±0.11, n=3; p= 0.0017, compared to WT GS 1 h
Fig. 1h	WT	Con	1±0, n=3
		GS 1 h	1.63±0.22, n=3
		GS 2 h	1.77±0.33, n=3
	KO	Con	0.90±0.19, n=3; p=0.9531, compared to WT con
		GS 1 h	1.00±0.25, n=3; p=0.0310, compared to WT GS 1 h
		GS 2 h	1.08±0.37, n=3; p=0.0186, compared to WT GS 2 h
Fig. 1k	WT	Con	1±0, n=3
		A23187	1.96±0.05, n=3; p= 0.0039, compared to WT Con
	KO	Con	1.04±0.44, n=3; p= 0.9811, compared to WT Con
		A23187	1.67±0.28, n=3; p= 0.0358, compared to KO Con; p= 0.3735, compared to WT Met.
Fig. 1i	WT	48 h	1.82±0.11, n=3
		72 h	1.22±0.08, n=3
		96 h	1.24±0.16, n=3
		120 h	1.27±0.08, n=3
	KO	48 h	0.87±0.13, n=3; p <0.0001, compared to WT 48 h
		72 h	0.75±0.07, n=3; p =0.0002, compared to WT 72 h
		96 h	0.76±0.10, n=3; p=0.0001, compared to WT 96 h
		120 h	0.50±0.06, n=3; p <0.0001, compared to WT 120 h
Fig. 1j	WT		0.69±0.02, n=3
	KO		0.44±0.09, n=3; p = 0.0090, compared to WT
Fig. if, 1g, 1h, 1k and 1i: p values calculated by two-way ANOVA, followed by Sidak's test; Fig. 1j: p values calculated by unpaired two-tailed t test, t=4.740, df=4. Normality Test for 1f,1h,1k,1i and 1j: normal distribution determined by Shapiro-Wilk test.			

Fig. 2			
Fig. 2a	Con		0.07±0.10, n = 79
	GS		0.42±0.25, n= 115; p <0.0001, compared to Con
Fig. 2b	Con		0.72±0.22, n= 55
	GS		0.70±0.23, n= 73; p= 0.9055, compared to Con
Fig. 2d	WT	Con	1.00±0.05, n = 33
		GS	1.27±0.12, n = 50; p <0.0001, compared to WT Con
	KO	Con	0.79±0.13, n = 40; p <0.0001, compared to WT Con
		GS	0.91±0.15, n = 41; p <0.0001, compared to WT GS; p <0.0001, compared to KO Con
Fig. 2e	WT	Con	1.00±0.17, n = 37
		GS	1.18±0.21, n = 58; p <0.0001, compared to WT Con
	KO	Con	1.03±0.18, n = 37; p =0.7394, compared to WT Con
		GS	1.13±0.19, n = 53; p =0.0345, compared to KO Con; p=0.2560, compared to WT GS
Fig. 2f	WT	Con	1±0, n=3
		Met	1.49±0.29, n=3; p = 0.0121, compared to WT Con
	KO	Con	1.00±0.07, n=3; p = 0.9998, compared to WT Con
		Met	1.37±0.14, n=3; p = 0.0452, compared to KO Con; p = 0.6312, compared to WT Met
Fig. 2h	WT	Con	1.00±0.51, n=35
		GS	3.76±3.98, n=34; p<0.001, compared to WT Con
	KO	Con	1.17±0.59, n=48; p=1.000, compared to WT Con
		GS	3.11±2.18, n=43; p<0.001, compared to KO Con; p=1.000, compared to WT GS
Fig. 2a and 2b: data without a normal distribution, p values calculated by unpaired two-tailed Mann Whitney test;<br>Fig. 2d, 2e and 2f: p values calculated by two-way ANOVA, followed by Sidak's test;<br>Fig. 2h: data without a normal distribution, p values calculated by Scheirer-Ray-Hare test;<br>Normality Test for 2d: normal distribution determined by Shapiro-Wilk test.<br>Normality Test for 2e: normal distribution determined by D'Agostino & Pearson test.			
Fig. 3			
Fig. 3d	WT		1±0, n=3
	KO		0.57±0.10, n=3; p=0.0016, compared to WT
Fig. 3e	WT		1±0, n=3
	KO		1.14±0.11, n=3; p=0.0948, compared to WT
Fig. 3f	WT	Con	1.00±0, n=3
		GS 1 h	0.97±0.10, n=3
		GS 2 h	1.34±0.21, n=3
	KO	Con	0.88±0.05, n=3; p=0.4563, compared to WT Con
		GS 1 h	0.89±0.01, n=3; p=0.7601, compared to WT GS 1 h

		GS 2 h	1.07±0.06, n=3; p=0.0205, compared to WT GS 2 h
Fig. 3h (left)	WT	Con	1.41±0.78, n=129
		Met	4.07±1.92, n=116; p <0.001, compared to WT Con
	KO	Con	1.44±0.84, n=120; p = 1.000, compared to WT Con
		Met	1.95±1.10, n=103; p=0.006, compared to KO Con; p <0.001, compared to WT Met
Fig. 3h (right)	WT	Con	0.36±0.32, n=129
		Met	0.95±0.60, n=116; p <0.001, compared to WT Con
	KO	Con	0.27±0.16, n=120; p=0.201, compared to WT Con
		Met	0.41±0.33, n=103; p= 0.003, compared to KO Con; p <0.001, compared to WT Met
Fig. 3d: p values calculated by unpaired two-tailed t test, t=7.589, df=4.<br>Fig. 3e: p values calculated by unpaired two-tailed t test, t=2.180, df=4.<br>Fig. 3f: p values calculated by two-way ANOVA, followed by Sidak's test;<br>Fig. 3h: data without a normal distribution, p values calculated by Scheirer-Ray-Hare test.<br>Normality Test for 3d,3e and 3f: normal distribution determined by Shapiro-Wilk test.			
Fig. 4			
Fig.4b	TBC1D23 FL		1±0, n=3
	△TBC		1.14±0.11, n=3; p=0.0766, compared to TBC1D23 FL
	△Rho		1.11±0.07, n=3; p=0.1636, compared to TBC1D23 FL
	TBC+ Rho		0.10±0.02, n=3; p<0.0001, compared to TBC1D23 FL
	514-684		1.09±0.06, n=3; p=0.3380, compared to TBC1D23 FL
Fig. 4c	514-684		1±0, n=3
	PH		0.94±0.12, n=3; p=0.4060, compared to 514-684
Fig. 4d	WT		1±0, n=3
	3K		0.24±0.16, n=3; p=0.0011, compared to WT
Fig. 4f	LKB1 FL		1±0, n=3
	△CRD		0.09±0.07, n=3; p<0.0001, compared to LKB1 FL
	△NRD		0.91±0.12, n=3; p=0.4129, compared to LKB1 FL
	KD		0.08±0.06, n=3; p<0.0001, compared to LKB1 FL
Fig. 4h	LKB1-CRD WT		1±0, n=3
	LKB1-CRD△LFa		0.21±0.09, n=3; p=0.0001, compared to LKB1-CRD WT
	LKB1-CRD 4K		0.33±0.14, n=3; p=0.0003, compared to LKB1-CRD WT
Fig. 4b,4f and 4h: p values calculated by one-way ANOVA, followed by Dunnett's test;<br>Fig. 4c: p values calculated by unpaired two-tailed t test, t=0.9279, df=4;<br>Fig. 4d: p values calculated by unpaired two-tailed t test, t=8.442, df=4.<br>Normality Test for 4b, 4c,4d,4f and 4h: normal distribution determined by Shapiro-Wilk test.			
Fig. 5			
Fig.5a	Con		1±0, n=3
	GS 0.5 h		1.81±0.50, n=3; p= 0.2634, compared to Con

	GS 1 h		2.52±0.61, n=3; p= 0.0196, compared to Con
	GS 2 h		2.44±0.77, n=3; p= 0.0265, compared to Con
	GS 4 h		1.38±0.48, n=3; p= 0.7991, compared to Con
Fig. 5b	Con		1±0, n=3
	GS 0.5 h		1.95±0.42, n=3; p= 0.0135, compared to Con
	GS 1 h		1.93±0.35, n=3; p= 0.0155, compared to Con
	GS 2 h		2.35±0.42, n=3; p= 0.0013, compared to Con
	GS 4 h		1.38±0.15, n=3; p= 0.4275, compared to Con
Fig. 5c (Con)	TBC1D23/LKB1		1±0, n=3
	TBC1D23/LKB1 AMPKα		1.77±0.12, n=3; p=0.0004, compared to TBC1D23/LKB1
Fig. 5d (GS)	TBC1D23/LKB1		1±0, n=3
	TBC1D23/LKB1 AMPKα		1.97±0.35, n=3; p=0.0090, compared to TBC1D23/LKB1
Fig. 5e (Con)	TBC1D23/LKB1 CRD		1±0, n=3
	TBC1D23/LKB1 CRD AMPKα		0.96±0.02, n=3; p=0.0646, compared to TBC1D23/LKB1 CRD
Fig. 5f (GS)	TBC1D23/LKB1 CRD		1±0, n=3
	TBC1D23/LKB1 CRD AMPKα		2.22±0.23, n=3; p=0.0007, compared to TBC1D23/LKB1 CRD
Fig. 5a and 5b: p values calculated by one-way ANOVA, followed by Dunnett's test;<br>Fig. 5c: p values calculated by unpaired two-tailed t test, t=11.23, df=4;<br>Fig. 5d: p values calculated by unpaired two-tailed t test, t=4.749, df=4;<br>Fig. 5e: p values calculated by unpaired two-tailed t test, t=2.531, df=4;<br>Fig.5f: p values calculated by unpaired two-tailed t test, t=9.322, df=4.<br>Normality Test for 5a-5f: normal distribution determined by Shapiro-Wilk test.			
Fig. 6			
Fig.6a	WT	Con	0.08±0.13, n=81
		GS	0.36±0.25, n=92; p <0.001, compared to WT Con;
	KO	Con	0.06±0.09, n=116; p=1.000, compared to WT Con
		GS	0.15±0.18, n=98; p <0.001, compared to WT GS; p <0.001, compared to KO Con
Fig.6b	Vector		1±0, n=3
	WT		1.68±0.34, n=3; p=0.0228, compared to Vector
	3K		1.03±0.23, n=3; p=0.9869, compared to Vector
	WT		1±0, n=3
	KO+Vector		0.70±0.01, n=3; p=0.9503, compared to WT

Fig. 6c	KO+LKB1	1.39±0.22, n=3; p=0.5732, compared to KO+Vector
	KO+LKB1-G WT	3.24±1.34, n=3; p=0.0037, compared to KO+Vector; p=0.0260, compared to KO+LKB1
	KO+LKB1-G D194A	1. 90±0.73, n=3; p=0.1691, compared to KO+Vector
Fig. 6e (upper)	WT	5.11±2.64, n=142
	KO+Vector	2.81±1.52, n=165; p<0.0001, compared to WT
	KO+LKB1	3.82±2.09, n=88; p= 0.0067, compared to KO+Vector
	KO+LKB1-G WT	10.30±4.12, n=79; p<0.0001, compared to KO+LKB1
	KO+LKB1-G D194A	4.33±2.91, n=85; p <0.0001, compared to KO+LKB1-G WT
Fig. 6e (lower)	WT	0.69±0.40, n=142
	KO+Vector	0.37±0.30, n=165; p<0.0001, compared to WT
	KO+LKB1	0.41±0.38, n=88; p >0.9999, compared to KO+Vector
	KO+LKB1-G WT	1.15±0.55, n=79; p<0.0001, compared to KO+LKB1
	KO+LKB1-G D194A	0.56±0.41, n=85; p<0.0001, compared to KO+LKB1-G WT
Fig. 6a: data without a normal distribution, p values calculated by Scheirer-Ray-Hare test; Fig. 6b and 6c: p values calculated by one-way ANOVA, followed by Dunnett's test; Fig. 6e: data without a normal distribution, p values calculated Kruskal-Wallis test. Normality Test for 6b and 6c: normal distribution determined by Shapiro-Wilk test.		
Fig. 7		
Fig.7b	Control	142.40±5.16, n=10
	TBC1D23 MO	93.08±14.57, n=11; p<0.0001, compared to Control
	MO+ TBC1D23	127.55±8.56, n=8; p<0.0001, compared to TBC1D23 MO
	MO+LKB1	100.01±15.04, n=12; p=0.4877, compared to TBC1D23 MO
	MO+LKB1-G WT	117.70±9.00, n=11; p<0.0001, compared to TBC1D23 MO
	MO+LKB1-G D194A	98.72±12.73, n=8; p=0.7559, compared to TBC1D23 MO
Fig. 7d	Control	10175.43±1176.97, n=14
	TBC1D23 MO	4057.78±950.51, n=13; p<0.0001, compared to Control
	MO+ TBC1D23	8463.90±1404.23, n=12; p<0.0001, compared to TBC1D23 MO
	MO+LKB1	4339.54±1248.08, n=15; p=0.9541, compared to TBC1D23 MO
	MO+LKB1-G WT	6423.06±1200.18, n=11; p<0.0001, compared to TBC1D23 MO
	MO+LKB1-G D194A	3860.55±1072.40, n=15; p=0.9900, compared to TBC1D23 MO
Fig. 7b and 7d: p values calculated by one-way ANOVA, followed by Dunnett's test.		

Normality Test for 7b and 7d: normal distribution determined by Shapiro-Wilk test.			
Fig. 8			
Fig.8a	WT		1±0, n=3
	F354L		0.83±0.08, n=3; p= 0.0278, compared to WT
	F354A		0.75±0.07, n=3; p= 0.0047, compared to WT
Fig. 8e	Control		143.20±7.82, n=11
	LKB1 MO		125.15±4.98, n=7; p=0.0069, compared to Control
	MO+LKB1 WT		139.90±7.17, n=10; p=0.9476, compared to Control; p=0.0374, compared to LKB1 MO
	MO+LKB1 F354L		119.01±10.04, n=6; p=0.0031, compared to MO+LKB1 WT
	MO+LKB1 F354A		114.27±21.30, n=7; p=0.0001, compared to MO+LKB1 WT
	MO+ TBC1D23		118.53±9.69, n=6; p=0.0024, compared to MO+LKB1 WT
Fig. 8g	Control		10435.59±1423.04, n=14
	LKB1 MO		5214.89±2749.37, n=18; p<0.0001, compared to Control
	MO+LKB1 WT		8285.97±2493.18, n=21; p= 0.2997 compared to Control; p= 0.0094, compared to LKB1 MO
	MO+LKB1 F354L		3780.29±2294.72, n=10; p=0.0004, compared to MO+LKB1 WT
	MO+LKB1 F354A		4202.38±3092.01, n=8; p=0.0116, compared to MO+LKB1 WT
	MO+ TBC1D23		3712.82±2697.75, n=8; p=0.0026, compared to MO+LKB1 WT
Fig. 8a, 8e: p values calculated by one-way ANOVA, followed by Dunnett's test.<br>Fig. 8g: data without a normal distribution, p values calculated by Kruskal-Wallis test.<br>Normality Test for 8a: normal distribution determined by Shapiro-Wilk test;<br>Normality Test for 8e: normal distribution determined by Kolmogorov-Smirnov test;			
Fig. S1			
Fig. S1h	WT	G1	64.37±2.02, n=3
		S	11.22±0.33, n=3
		G2/M	24.04±2.31, n=3
	KO	G1	65.80±0.95, n=3; p= 0.5863, compared to WT G1
		S	11.15±1.45, n=3; p >0.9999, compared to WT S
		G2/M	22.42±0.65, n=3; p= 0.4875, compared to WT G2/M

Fig. S1i	WT	48 h	2.51±0.07, n=3
		72 h	4.32±0.20, n=3
	KO	48 h	2.63±0.03, n=3; p= 0.5591, compared to WT 48 h
		72 h	4.26±0.17, n=3; p= 0.8482, compared to WT 72 h
Fig. S1h and 1i: p values calculated by two-way ANOVA, followed by Sidak's test.<br>Normality Test for S1h and S1i: normal distribution determined by Shapiro-Wilk test.			
Fig. S3			
Fig. S3a	Con		1.00±0.17, n=23
	GS		1.00±0.14, n=23; p >0.9999, compared to Con
Fig. S3b	Con		1.00±0.13, n=37
	GS		1.00±0.10, n=38; p= 0.8658, compared to Con
Fig. S3c	Vector	Con	1±0, n=3
		AICAR	1.23±0.15, n=3
	LKB1	Con	1.42±0.08, n=3; p= 0.0139, compared to Vector Con
		AICAR	1.69±0.23, n=3; p= 0.0093, compared to Vector AICAR
Fig. S3d	Vector	Con	1±0, n=3
		AICAR	0.93±0.04, n=3
	LKB1	Con	1.32±0.18, n=3; p= 0.0129, compared to Vector Con
		AICAR	1.56±0.11, n=3; p= 0.0002, compared to Vector AICAR
Fig. S3e	WT	Con	1±0, n=3
		0.5 mM Met	1.38±0.16, n=3
		1 mM Met	1.35±0.10, n=3
	KO	Con	1.14±0.16, n=3; p= 0.7618, compared to WT Con
		0.5 mM Met	1.34±0.18, n=3; p= 0.9875, compared to WT 0.5 mM Met
		1 mM Met	1.46±0.35, n=3; p= 0.8664, compared to WT 1 mM Met
Fig. S3a: p values calculated by unpaired two-tailed t test, t=9.444e-008, df=44;<br>Fig. S3b: p values calculated by unpaired two-tailed t test, t=0.1696, df=73;<br>Fig. S3c, S3d and S3e: p values calculated by two-way ANOVA, followed by Sidak's test.<br>Normality Test for S3a: normal distribution determined by Kolmogorov-Smirnov test;<br>Normality Test for S3b, S3c, S3d and S3e: normal distribution determined by Shapiro-Wilk test.			
Fig. S6			

Fig. S6c	GFP-N1	0.71±0.05, n=27
	GFP-CRD	0.62±0.08, n=42; p <0.0001, compared to GFP-N1
Fig. S6f	Con	0.75±0.09, n=72
	GS	0.69±0.11, n=84; p =0.0018, compared to Con
	Met	0.62±0.12, n=86; p <0.0001, compared to Con
Fig. S6h	Con	0.34±0.12, n=69
	GS	0.19±0.10, n=63; p <0.0001, compared to Con
	Met	0.15±0.10, n=66; p <0.0001, compared to Con
Fig. S6c: p values calculated by unpaired two-tailed t test, t=5.317, df=67;<br>Fig. S6f and S6h: p values calculated by one-way ANOVA, followed by Dunnett's test.<br>Normality Test for S6c: normal distribution determined by Shapiro-Wilk test;<br>Normality Test for S6f and S6h: normal distribution determined by Kolmogorov-Smirnov test.		
Fig. S7		
Fig. S7	WT	1.00±0.03, n=3
	LKB1 MO 2 ng	0.28±0.04, n=3; p=0.9245, compared to WT
	LKB1 MO 5 ng	0.09±0.002, n=3; p=0.0067, compared to WT
	LKB1 MO 7 ng	0.11±0.01, n=3; p=0.1246, compared to WT
data without a normal distribution (LKB1 MO 7 ng group failed to pass normality test), p values calculated by Kruskal-Wallis test.		
