# Supplementary material for: TBC1D23 mediates Golgi-specific LKB1 signaling
Source: Nat Commun. 2024 Feb 27;15:1785. doi: 10.1038/s41467-024-46166-2 (PMC10899256; doi:10.1038/s41467-024-46166-2)
Supplement: Supplementary file 3 — Description of Additional Supplementary Files [file 41467_2024_46166_MOESM3_ESM.pdf]

Description of Additional Supplementary Files

File Name: Supplementary Data 1

Description: List of TBC1D23-interacting proteins identified by mass spectrometry. HEK293T cells were transfected with GST-vector or GST-TBC1D23. The proteins associated specifically with GST-tagged TBC1D23 versus GST were identified by mass spectrometry.

File Name: Supplementary Data 2

Description: Differential phosphosites lists. WT and TBC1D23 knockout HEK293T cells glucose starved for 2 h were subjected to quantitative phosphoproteomic analysis by mass spectrometry. p values were determined by unpaired two-tailed t test

Sheet Downregulated in sgTBC1D23: 103 phosphosites with higher phosphorylation levels in WT HEK293T cells than in TBC1D23 KO cells.

Sheet Upregulated in sgTBC1D23: 40 phosphosites with higher phosphorylation levels in TBC1D23 KO HEK293T cells than in WT cells.
