# Supplementary material for: TBC1D23 mediates Golgi-specific LKB1 signaling
Source: Nat Commun. 2024 Feb 27;15:1785. doi: 10.1038/s41467-024-46166-2 (PMC10899256; doi:10.1038/s41467-024-46166-2)
Supplement: Supplementary file 6 — Reporting Summary [file 41467_2024_46166_MOESM6_ESM.pdf]

nature portfolio

Corresponding author(s): Da Jia
Last updated by author(s): Jan 31, 2024

Reporting Summary

Nature Portfolio wishes to improve the reproducibility of the work that we publish. This form provides structure for consistency and transparency in reporting. For further information on Nature Portfolio policies, see our Editorial Policies and the Editorial Policy Checklist.

Statistics

For all statistical analyses, confirm that the following items are present in the figure legend, table legend, main text, or Methods section.

n/a	Confirmed	
☐	☒	The exact sample size (*n*) for each experimental group/condition, given as a discrete number and unit of measurement
☐	☒	A statement on whether measurements were taken from distinct samples or whether the same sample was measured repeatedly
☐	☒	The statistical test(s) used AND whether they are one- or two-sided<br>*Only common tests should be described solely by name; describe more complex techniques in the Methods section.*
☒	☐	A description of all covariates tested
☒	☐	A description of any assumptions or corrections, such as tests of normality and adjustment for multiple comparisons
☐	☒	A full description of the statistical parameters including central tendency (e.g. means) or other basic estimates (e.g. regression coefficient) AND variation (e.g. standard deviation) or associated estimates of uncertainty (e.g. confidence intervals)
☐	☒	For null hypothesis testing, the test statistic (e.g. *F*, *t*, *r*) with confidence intervals, effect sizes, degrees of freedom and *P* value noted<br>*Give P values as exact values whenever suitable.*
☒	☐	For Bayesian analysis, information on the choice of priors and Markov chain Monte Carlo settings
☒	☐	For hierarchical and complex designs, identification of the appropriate level for tests and full reporting of outcomes
☒	☐	Estimates of effect sizes (e.g. Cohen's *d*, Pearson's *r*), indicating how they were calculated

Our web collection on statistics for biologists contains articles on many of the points above.

Software and code

Policy information about availability of computer code

Data collection	There is no previously unreported custom computer code or algorithm used in this manuscript.<br>SAGECREATION was used to acquire western blot images.<br>Olympus FV3000 was used for confocal imaging of cell lines, and Zeiss ZEN was used for confocal imaging of iPSC-derived neurons and zebrafish. Confocal images of zebrafish midbrain were acquired by Olympus IX83 P2ZF.<br>MS : Q Exactive Plus for identification of TBC1D23-interacting proteins; Q Exactive HF-X for phosphoproteomic data collection.
Data analysis	Statistical analysis: GraphPad Prism 8; Image analysis: ImageJ (Confocal microscopy and Western blot) and ZEN (zebrafish); MS: MaxQuant version 1.6.2.3. And Scheirer-Ray-Hare Test was performed using SPSS 22 (IBM). For Z-ztacks of zebrafish embryos, deconvolution analysis using TRUSIGHT (cellSens Dimension, Olympus) and maximum intensity projection using MAX-Z (cellSens Dimension, Olympus) were applied to improve the contrast and resolution of raw images.

For manuscripts utilizing custom algorithms or software that are central to the research but not yet described in published literature, software must be made available to editors and reviewers. We strongly encourage code deposition in a community repository (e.g. GitHub). See the Nature Portfolio guidelines for submitting code & software for further information.

Data

Policy information about availability of data

All manuscripts must include a data availability statement. This statement should provide the following information, where applicable:

- Accession codes, unique identifiers, or web links for publicly available datasets
- A description of any restrictions on data availability
- For clinical datasets or third party data, please ensure that the statement adheres to our policy

Source data are provided with this paper. The mass spectrometry raw data of Fig.1 and Fig.3 were deposited to the ProteomeXchange Consortium (http://proteomecentral.proteomexchange.org) via the iProx partner repository,with data set identifier IPX0006072000. Raw MS data were analyzed by MaxQuant (version 1.6) and queried against the Swiss-Prot human protein sequence database. All the other data supporting the findings of this study are available within the article and its supplementary information files and from the corresponding author upon reasonable request.

Research involving human participants, their data, or biological material

Policy information about studies with human participants or human data. See also policy information about sex, gender (identity/presentation), and sexual orientation and race, ethnicity and racism.

Reporting on sex and gender	No human participants used in this study.
Reporting on race, ethnicity, or other socially relevant groupings	No human participants used in this study.
Population characteristics	No human participants used in this study.
Recruitment	No human participants used in this study.
Ethics oversight	No human participants used in this study.

Note that full information on the approval of the study protocol must also be provided in the manuscript.

Field-specific reporting

Please select the one below that is the best fit for your research. If you are not sure, read the appropriate sections before making your selection.

☒ Life sciences ☐ Behavioural & social sciences ☐ Ecological, evolutionary & environmental sciences

For a reference copy of the document with all sections, see nature.com/documents/nr-reporting-summary-flat.pdf

Life sciences study design

All studies must disclose on these points even when the disclosure is negative.

Sample size	Sample size calculations were not preformed. Sample sizes were determined according to previous study published by us (Nat Commun. 2021 May 31;12(1):3258; Nat Struct Mol Biol. 2021 May;28(5):1-12.) and others using similar biological samples and techniques to detect the significant difference (Nat Commun. 2023 Nov 21;14(1):7598.). All experiments were performed using at least 3 independent biological repeats. See specific legends for details.
Data exclusions	No data was excluded from the analysis.
Replication	All experiments were performed using at least 3 independent biological repeats. And similar results were obtained.
Randomization	Experimental groups were defined based on appropriate biological and technical controls.
Blinding	The investigators were not blinded to all conditions as they were responsible for both experimental design and data collection. And we use blind image analysis for counting Golgi fragments. No subjective assessments were made.

Reporting for specific materials, systems and methods

We require information from authors about some types of materials, experimental systems and methods used in many studies. Here, indicate whether each material, system or method listed is relevant to your study. If you are not sure if a list item applies to your research, read the appropriate section before selecting a response.

Materials & experimental systems

n/a	Involved in the study
☐	☒ Antibodies
☐	☒ Eukaryotic cell lines
☒	☐ Palaeontology and archaeology
☐	☒ Animals and other organisms
☒	☐ Clinical data
☒	☐ Dual use research of concern
☒	☐ Plants

Methods

n/a	Involved in the study
☒	☐ ChIP-seq
☒	☐ Flow cytometry
☒	☐ MRI-based neuroimaging

Antibodies

Antibodies used

Antibodies used in this study were obtained from commercial resources: rabbit polyclonal anti-TBC1D23 (Proteintech, 17002-1-AP, Western blot (WB) 1:1,000), rabbit anti-LKB1(Cell Signaling Technology, 3047, WB 1:1,000), rabbit anti-FAM21(donated by Dr. Daniel D. Billadeauh, WB 1:1,000), rabbit anti-AMPKα (Cell Signaling Technology, 2532, WB 1:1,000), rabbit anti-phospho-AMPKα-Thr172 (Cell Signaling Technology, 2535, WB 1:1,000), mouse anti-phospho-CaMKII-Thr286 (abcam, ab171095, WB 1:1,000), rabbit anti-Flag (Proteintech, 20543-1-AP, WB 1:2,000), mouse anti-Flag (Sigma-Aldrich, F1804, WB 1:2,000, IF 1:300), rabbit anti-golgin-97 (Proteintech, 12640-1-AP, IF 1:200, WB 1:1000), mouse anti-LAMP1 (abcam, ab289548, WB 1:1000), mouse anti-TOM20 (santa cruz, sc-17764, WB 1:500), rat anti-HA (Roche, 11867423001,WB 1:2000), rabbit anti-GST (Proteintech, 10000-0-AP, WB 1:2,000), mouse anti-GFP (Proteintech, 66002-1-Ig, WB 1:2,000), rabbit anti-GBF1(Proteintech, 25183-1-AP, WB 1:1,000), rabbit anti-phospho-GBF1-Thr1337, (Immuno-Biological lab, 28065, WB 5 μg/mL), rabbit anti-GAPDH (Proteintech, 10494-1-AP, WB 1:2,000), rabbit anti-beta actin (ABclonal, AC026, WB 1:2,000), rabbit anti-mCherry (Proteintech, 26765-1-AP, WB 1:2,000), rabbit anti- Phospho-(Ser/Thr) (Cell Signaling Technology, 9631, WB 1:1,000), rabbit anti-GM130 (Abcam, 52649, IF 1:300), mouse anti-GM130 (BD, 610822, IF 1:300), rabbit anti-ZFPL1 (Invitrogen, PA5-53254, IF 1:300), mouse anti-TGN46 (Abcam, 50595, IF 1:300) and mouse anti-CIMPR (Bio-Rad, MCA2048, IF 1:300). Goat anti-Rabbit IgG Secondary Antibody HRP conjugated (SAB, L3012-2), Goat anti-Mouse IgG Secondary Antibody HRP conjugated (SAB, L3032-2). HRP-conjugated Affinipure Goat anti-Rat IgG(H+L) (Proteintech, SA00001-15). Alexa-labelled secondary antibodies were from Invitrogen (1:2000).

Validation

Antibodies validation was either through the manufacture's validation sheet (see detailed information above for the precise manufacture and ID) or published validation by other research groups:
1. rabbit polyclonal anti-TBC1D23 (Proteintech, 17002-1-AP)/Western blot
   https://www.ptgcn.com/Products/TBC1D23-Antibody-17002-1-AP.htm
   Huang, W. et al. Proc Natl Acad Sci U S A. 2019 Nov 5;116(45):22598-22608. doi: 10.1073/pnas.1909316116.
2. rabbit anti-LKB1(Cell Signaling Technology, 3047)/Western blot
   https://www.cellsignal.com/products/primary-antibodies/lkb1-d60c5-rabbit-mab/3047
   Zhang, Y.L. et al. Cell Metab. 2013 Oct 1;18(4):546-55. doi: 10.1016/j.cmet.2013.09.005.
3. rabbit anti-FAM21(donated by Dr. Daniel D. Billadeauh)/Western blot
   Huang, W. et al. Proc Natl Acad Sci U S A. 2019 Nov 5;116(45):22598-22608. doi: 10.1073/pnas.1909316116.
4. rabbit anti-AMPKα (Cell Signaling Technology, 2532)/Western blot
   https://www.cellsignal.com/products/primary-antibodies/ampka-antibody/2532
   Zhang, Y.L. et al. Cell Metab. 2013 Oct 1;18(4):546-55. doi: 10.1016/j.cmet.2013.09.005.
5. rabbit anti-phospho-AMPKα-Thr172 (Cell Signaling Technology, 2535)/Western blot
   https://www.cellsignal.com/products/primary-antibodies/phospho-ampka-thr172-40h9-rabbit-mab/2535
   Zhang, Y.L. et al. Cell Metab. 2013 Oct 1;18(4):546-55. doi: 10.1016/j.cmet.2013.09.005.
6. mouse anti-phospho-CaMKII-Thr286 (abcam, ab171095)/Western blot
   https://www.abcam.com/products/primary-antibodies/camkii-phospho-t286-antibody-22b1-ab171095.html
   Bhattacharyya M et al. Elife. 2020 Mar 9:9:e53670. doi: 10.7554/eLife.53670.
7. rabbit anti-Flag (Proteintech, 20543-1-AP)/Western blot
   https://www.ptgcn.com/products/Flag-Tag-Antibody-20543-1-AP.htm
   Yang Z.J. et al. Nat Metab. 2023 Jan;5(1):61-79. doi: 10.1038/s42255-022-00710-w.
8. mouse anti-Flag (Sigma-Aldrich, F1804)/Western blot, Immunofluorescence
   https://www.sigmaaldrich.cn/CN/zh/product/sigma/f1804
   Monika Srivastava et al. Nat Commun. 2015 Feb 20:6:6253. doi: 10.1038/ncomms7253.
9. rabbit anti-golgin-97 (Proteintech, 12640-1-AP)/Western blot, Immunofluorescence
   https://www.ptgcn.com/products/GOLGA1-Antibody-12640-1-AP.htm
   Lucía Barbier-Torres et al. Nat Commun. 2022 Jan 28;13(1):557. doi: 10.1038/s41467-022-28201-2.
   Zhang X.Y. et al. Dev Cell. 2018 Apr 23;45(2):245-261.e6. doi: 10.1016/j.devcel.2018.03.023.
10. mouse anti-LAMP1 (abcam, ab289548)/Western blot
    https://www.abcam.com/products/primary-antibodies/lamp1-antibody-25lamp-1-ab289548.html
11. mouse anti-TOM20 (santa cruz, sc-17764)/Western blot
    https://www.scbt.com/p/tom20-antibody-f-10
    Brix, J., et al. 1999. J Biol Chem. 274: 16522-30. doi: 10.1074/jbc.274.23.16522.
12. rat anti-HA (Roche, 11867423001)/Western blot
    https://www.sigmaaldrich.cn/CN/zh/product/roche/roahaha
    Kristina Halbleib et al. doi: 10.1016/j.molcel.2017.06.012.
13. rabbit anti-GST (Proteintech, 10000-0-AP)/Western blot
    https://www.ptgcn.com/products/gst-Antibody-10000-0-AP.htm
    Xiao J. et al. Immunity. 2020 Jan 14;52(1):109-122.e6. doi: 10.1016/j.immuni.2019.11.015.
14. mouse anti-GFP (Proteintech, 66002-1-Ig)/Western blot
    https://www.ptgcn.com/products/eGFP-Antibody-66002-1-Ig.htm

Zhang X.Y. et al. Cell Discov. 2023 Jul 11;9(1):71. doi: 10.1038/s41421-023-00551-1.
15. rabbit anti-GBF1(Proteintech, 25183-1-AP)/Western blot
https://www.ptgcn.com/products/GBF1-Antibody-25183-1-AP.htm
Wang T. et al. PLoS Pathog. 2017 Oct 6;13(10):e1006674. doi: 10.1371/journal.ppat.1006674.
16. rabbit anti-phospho-GBF1-Thr1337, (Immuno-Biological lab, 28065)/Western blot
https://www.ibl-japan.co.jp/en/search/product/detail/id=3818
Mafalda Lopes-da-Silva et al. Dev Cell. 2019 Jun 3;49(5):786-801.e6. doi: 10.1016/j.devcel.2019.04.006.
17. rabbit anti-GAPDH (Proteintech, 10494-1-AP)/Western blot
https://www.ptgcn.com/products/GAPDH-Antibody-10494-1-AP.htm
Qiu S.Q. et al. Cell Res. 2023 Apr;33(4):299-311. doi: 10.1038/s41422-023-00788-1.
18. rabbit anti-beta actin (ABclonal, AC026)/Western blot
https://abclonal.com.cn/catalog/AC026
Chen B. et al. Nature. 2022 May;605(7911):761-766. doi: 10.1038/s41586-022-04756-4.
19. rabbit anti-mCherry (Proteintech, 26765-1-AP)/Western blot
https://www.ptgcn.com/products/mCherry-Antibody-26765-1-AP.htm
Cheng X.X. et al. Autophagy. 2023 Jan;19(1):241-255. doi: 10.1080/15548627.2022.2071381.
20. rabbit anti- Phospho-(Ser/Thr) (Cell Signaling Technology, 9631)/Western blot
https://www.cellsignal.com/products/primary-antibodies/phospho-ser-thr-phe-antibody/9631
Wang X.Y. et al. Nat Commun. 2023 Mar 2;14(1):1185. doi: 10.1038/s41467-023-36865-7.
21. rabbit anti-GM130 (Abcam, 52649)/Immunofluorescence
https://www.abcam.com/products/primary-antibodies/gm130-antibody-ep892y-cis-golgi-marker-ab52649.html
Robert Mahen. PLoS Biol. 2022 Oct 25;20(10):e3001854. doi: 10.1371/journal.pbio.3001854.
22. mouse anti-GM130 (BD, 610822)/Immunofluorescence
https://www.bdbiosciences.com/en-us/products/reagents/microscopy-imaging-reagents/immunofluorescence-reagents/purified-mouse-anti-gm130.610822
P Marra et al. Nat Cell Biol. 2001 Dec;3(12):1101-13. doi: 10.1038/ncb1201-1101.
23. rabbit anti-ZFPL1 (Invitrogen, PA5-53254)/Immunofluorescence
https://www.thermofisher.cn/cn/zh/antibody/product/ZFPL1-Antibody-Polyclonal/PA5-53254
Liu D.D. et al. PLoS Biol. 2020 May 26;18(5):e3000746. doi: 10.1371/journal.pbio.3000746.
24. mouse anti-TGN46 (Abcam, 50595)/Immunofluorescence
https://www.abcam.com/products/primary-antibodies/tgn46-antibody-ab50595.html
Guo Z et al. Cell Rep. 2022 Mar 1;38(9):110452. doi: 10.1016/j.celrep.2022.110452.
25. mouse anti-CIMPR (Bio-Rad, MCA2048)/Immunofluorescence
https://www.bio-rad-antibodies.com/monoclonal/human-cd222-antibody-mem-238-mca2048.html
Hertel, A. et al. Cell Rep. 2022 Dec 6;41(10):111653. doi: 10.1016/j.celrep.2022.111653.
26. Goat anti-Rabbit IgG Secondary Antibody HRP conjugated (SAB, L3012-2)/Western blot
https://www.sabbiotech.com.cn/g-170627-Goat-anti-Rabbit-IgG-Secondary-AntibodyHRP-conjugated-L3012.html
Han Z et al. Nat Commun. 2021 May 31;12(1):3258. doi: 10.1038/s41467-021-23539-5.
27. Goat anti-Mouse IgG Secondary Antibody HRP conjugated (SAB, L3032-2)/Western blot
https://www.sabbiotech.com.cn/g-170611-Goat-anti-Mouse-IgG-Secondary-AntibodyHRP-conjugated-L3032.html
Han Z et al. Nat Commun. 2021 May 31;12(1):3258. doi: 10.1038/s41467-021-23539-5.
28. HRP-conjugated Affinipure Goat Anti-Rat IgG(H+L) (Proteintech, SA00001-15)/Western blot
https://www.ptgcn.com/products/HRP-conjugated-Affinipure-Goat-Anti-Rat-IgG-H-L-secondary-antibody.htm
Ding WL et al. Nat Commun. 2020 Jun 22;11(1):3154. doi: 10.1038/s41467-020-16898-y.
29. Alexa-labelled secondary antibodies were from Invitrogen/Immunofluorescence
https://www.thermofisher.cn/antibody/secondary/query/*

Eukaryotic cell lines

Policy information about cell lines and Sex and Gender in Research

Cell line source(s)	HEK293T, HeLa, A549, and NIH 3T3 were obtained from ATCC, and HepG2 was obtained from Jennio Biotech.
Authentication	The cells were validated by STR profiling.
Mycoplasma contamination	All cell lines were routinely verified to be free of mycoplasma contamination.
Commonly misidentified lines (See ICLAC register)	No commonly misidentified lines were used in this study.

Animals and other research organisms

Policy information about studies involving animals; ARRIVE guidelines recommended for reporting animal research, and Sex and Gender in Research

Laboratory animals	The following zebrafish lines were used in this study: AB strain (wild-type), Tg[HuC:GFP] strain, and Tg[Hb9:GFP]ml2 strain. The MO and mRNAs were injected into the yolk and the cell at the one-cell stage in all zebrafish experiments. Statistical measurements were performed at 48 hours post fertilization (hpf) across all zebrafish strains utilized in this study.
Wild animals	Not used.
Reporting on sex	For zebrafish, sex was not determined throughout the experiment.

Field-collected samples | Not used.

Ethics oversight | The animal welfare and experiments conformed to the guidelines for care and use of laboratory animals, and were performed according to the guidelines and approval of the Animal Investigation Committee of the West China Second University Hospital, Sichuan University.

Note that full information on the approval of the study protocol must also be provided in the manuscript.

Plants

Seed stocks | None

Novel plant genotypes | None

Authentication | None
